# Acetylcholinesterase (*Ace-1*) target site mutation 119S is strongly diagnostic of carbamate and organophosphate resistance in *Anopheles gambiae s.s. and Anopheles coluzzii* across southern Ghana

**DOI:** 10.1186/1475-2875-12-404

**Published:** 2013-11-09

**Authors:** John Essandoh, Alexander E Yawson, David Weetman

**Affiliations:** 1 Liverpool School of Tropical Medicine, Liverpool, UK; 2 Biotechnology & Nuclear Agriculture Research Institute, Ghana Atomic Energy Commission, Accra, Ghana; 3 Department of Entomology and Wildlife, University of Cape Coast, Cape Coast, Ghana

**Keywords:** Mosquito, Insecticide resistance, Diagnostic marker, Gene duplication

## Abstract

**Background:**

With high DDT resistance present throughout much of West Africa, carbamates and organophosphates are increasingly important alternatives to pyrethroids for indoor residual spraying (IRS). Though less widespread, resistance to both of these alternative insecticide classes has also been documented within the *Anopheles gambiae* species pair (formerly the M and S molecular forms) in West Africa. To manage insecticide efficacy, it is important to predict how and where resistance is likely to occur and spread, which could be aided by using molecular diagnostics with high predictive value.

**Methods:**

*Anopheles coluzzii* and *An. gambiae s.s.* were collected from 18 sites throughout southern Ghana and bioassayed with bendiocarb, the most commonly applied carbamate, and an organophosphate, fenitrothion. The *Ace-1* target site substitution G119S was genotyped by qPCR.

**Results:**

Fenitrothion induced higher mortality than bendiocarb, though phenotypes correlated strongly across populations. *Ace-1* 119S was found at much higher frequency in *An. gambiae s.s* than *An. coluzzii*, exceeding 90% in a population from Greater Accra, the highest frequency reported to date. *Ace-1* G119S was very strongly associated with resistance to both insecticides, providing high predictive power for diagnosis, though with some evidence for a differential effect between molecular forms for bendiocarb. Sequencing of the gene revealed a lack of variation in resistant alleles precluding determination of origin, but *Ace-1* copy number variation was detected for the first time in Ghana.

**Conclusions:**

The results validate G119S as a useful diagnostic of organophosphate and carbamate resistance within and among populations, whilst highlighting the potential for an aggregate nature of *Ace-1* genotypes, which may comprise both single-copy and duplicated genes. Further work is now required to determine the distribution and resistance-association of *Ace-1* duplication.

## Background

Safe and effective deployment of insecticides via indoor residual spraying (IRS), treated bed nets (ITNs) and more recently long-lasting insecticidal nets (LLINs) have been key elements contributing to recent reductions in malaria in sub-Saharan Africa [1]. Only pyrethroids are currently used for ITNs/LLINs and, alongside DDT, have been most widely used for IRS [2]. In West Africa, DDT and pyrethroid resistance in both *Anopheles coluzzii* and *An. gambiae s.s.* respectively [3]), is widespread and frequently co-occurs [4]. Covariation of DDT and pyrethroid resistance is at least partially attributable to reductions in insecticide efficacy caused by point mutations in their common target site, the voltage-gated sodium channel (*Vgsc*) [5-7], and perhaps also some sharing of metabolic resistance mechanisms [8]. To sustain the efficacy of IRS and maintain or recover the efficacy of pyrethroids for ITNs, it is essential to increase the application of insecticides with a different mode of action, for example in spatial combination or temporal rotations.

Carbamates and organophosphates are currently the available alternatives for adult vector control. Experimental hut studies have shown that organophosphates and carbamates can be effective against pyrethroid–resistant *An. gambiae*[9], and the carbamate bendiocarb has been applied successfully for IRS in several countries [10-12], with organophosphate-based IRS likely to increase. Carbamates and organophosphates are also prone to impact by mutations in their shared target site, acetycholinesterase, encoded by *Ace-1* in *An. gambiae*. A point substitution, *Ace-1* G119S, has been linked directly or indirectly to insecticide survivorship in laboratory strains [13,14] and field studies [15,16], and is the only known resistance-associated substitution in *An. gambiae*. However, more information on the importance of G119S for carbamate and organophosphate resistance across wild populations of *Anopheles* is required, in order to its evaluate diagnostic capacity, and the strength of cross-resistance requires [14].

In Ghana, malaria is hyperendemic and a major cause of low productivity and poverty. Data on insecticide resistance are patchy, but where studied in central and southern Ghana, prevalence of pyrethroid and DDT resistance within populations appears high [17-20] and carbamate resistance has been detected [17,20]. Southern Ghana comprises of five ecological zones, which have been linked to population discontinuities in the *An. gambiae* species pair [21]. Major genetic discontinuity is also present between the constituent species of the pair *An. coluzzii* and *An. gambiae s.s.*[19,22], but hybridization does occur at a low rate [22] and has proved consequential in permitting transfer and spread of the insecticide resistance mutation *Vgsc* 1014 F (formerly termed *kdr* west) [19,23,24]. At present, information on the origin and spread of the *An. gambiae Ace-1* 119S allele is entirely lacking in Ghana and elsewhere.

Information on patterns of insecticide resistance, occurrence of multiple resistance, and mechanisms of cross-resistance are vital to permit rational deployment and management of the four insecticide classes available for malaria control [25]. Moreover, understanding and prediction of the origins and spread of resistance would be greatly enhanced by robust molecular diagnostics, which requires evaluation in field populations. The present study aimed to characterize patterns of carbamate and organophosphate resistance across southern Ghana and to evaluate the predictive power of the *Ace-1* G119S substitution as a molecular resistance diagnostic. The results show that much of the considerable variance in resistance is attributable to variation in G119S, demonstrating its utility as the best currently available molecular resistance-diagnostic for wild *Anopheles*.

## Methods

### Sampling

**Figure 1:**
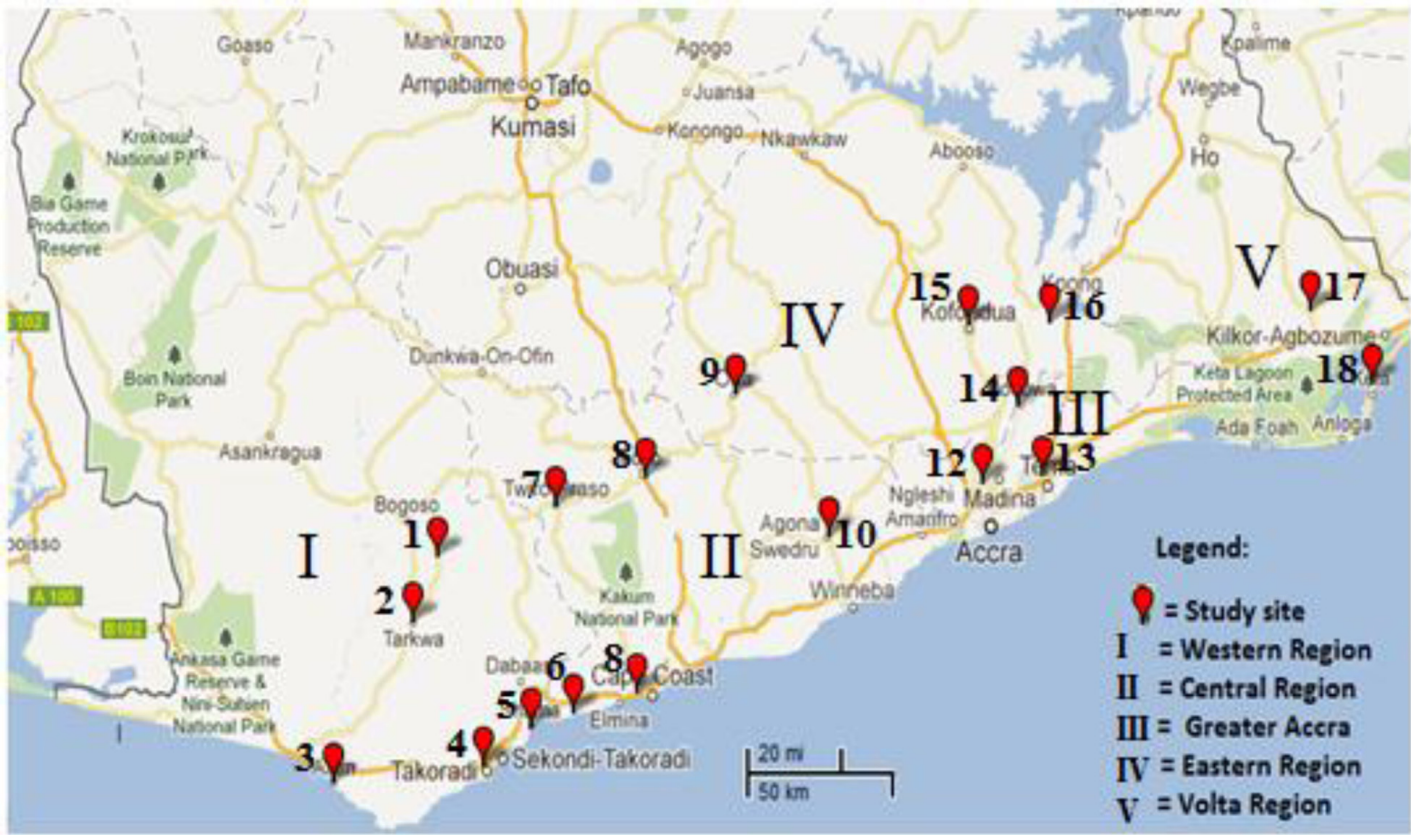
Map of southern Ghana showing the study sites (1-18) across administrative regions (I-V).

Mosquitoes were sampled from 18 sites spanning five distinct ecological zones in southern Ghana (Figure 1). Study sites were classified qualitatively based on settlement (rural, peri-urban and urban), and specific agricultural activities (irrigation, semi-irrigation and dry cultivation) that lead to creation of mosquito breeding sites as well as deployment of agricultural insecticides (Additional file 1). *Anopheles gambiae* larvae were collected from early March to mid-August 2011 from urban, peri-urban and rural settings. The larvae were collected from a variety of habitats such as pools, puddles, drainage channels, irrigations, and rice fields by the dipping method. Larvae were fed with ground Tetramin fish-food and upon pupation were transferred to a cage to emerge into adults. Cotton wool pads soaked with 10 % sugar solution were used to feed the adult mosquitoes. Male mosquitoes were removed from cages daily so as to avoid mating.

#### Bioassays

Approximately 25 three to five-day old adult females were aspirated into WHO bioassay tubes and exposed to either 0.1% bendiocarb-treated or 1.0% fenitrothion-treated papers for one hour. It should be noted that this followed the WHO protocol for both insecticides when the experiments were conducted, but the exposure time for fenitrothion recommended by WHO has subsequently been amended to two hours [25]. Four replicate bioassay tubes were run, along with a control tube containing paper-lacking insecticide. Following the exposure period, females were gently transferred back to holding tubes and supplied with 10% sucrose solution. Mortality was recorded after a 24-hour period, with knockdown at 24 hours considered dead. Dead and live mosquitoes were counted and stored separately in perforated, labelled 0.2-ml Eppendorf tubes and preserved using silica gel for molecular analysis. Where control mortalities exceeded 5%, Abbott’s formula [26] was used to correct non-insecticide-induced mortality. Susceptibility status was determined according to WHO criteria [25], though for comparison with future studies the one hour exposure used for fenitrothion should be noted.

### Identification of species and *Ace-1* genotype

Forty-eight bioassayed mosquitoes from each study site were selected randomly for sibling species complex determination using a standard PCR protocol [27] though using a leg from each mosquito as template, rather than pre-extracted DNA. PCR products were visualized under ultraviolet (UV) light after electrophoresis using 2% agarose gel stained with ethidium bromide. From individual females confirmed as belonging to the *An. gambiae s.s.* species pair, total genomic DNA was extracted using QIAGEN DNeasy 96-kits according to the manufacturer’s instructions, and stored at 4°C. Identification of species within *An. gambiae s.s.* (i.e. M or S molecular form) was determined using the SINE PCR protocol [28]. DNA extracts of mosquitoes of known species were genotyped individually using a TaqMan assay [29], run on an Agilent Stratagene MX3000 qPCR thermal cycler. Genotypes were scored from bi-directional scatter plots produced by the Agilent MxPro software after amplification.

#### *Ace-1* sequencing

In order to investigate polymorphism in the *Ace-1* gene which might provide information on the origin of resistant alleles, 19 serine homozygotes and one glycine homozygote (identified by TaqMan assay) were selected for sequencing. A 924-bp genomic DNA fragment, encompassing exons 4–6 (VectorBase AgamP3 annotation; G119S position is in exon 5) was amplified in a total volume of 50 μl containing 10 picomoles of each primer Ex2Agdir1 (5’ AGG TCA CGG TGA GTC CGT ACG A 3’) and Ex4Agrev2 (5’ AGG GCG GAC AGC AGA TGC AGC GA 3’), 10 mM dNTPs, ddH_**2**_O, 5X HF Phusion buffer, and 1u of Phusion Taq polymerase (Fermentas). PCR conditions were an initial denaturation step of 4 min at 98°C, followed by 35 cycles of 98°C for 30 sec, 64°C for 15 sec and 72°C for 30 sec, with final extension at 72°C for 5 min. Amplicons were visualized by gel electrophoresis using 2% agarose stained with ethidium bromide. Amplicons were purified using Fermentas GeneJET™ PCR Purification Kit in accordance with the manufacturer’s instructions. Amplicons were sequenced directly using the primers Ex2Agdir1 and Ex4Agrev2 by Macrogen (Korea) using the service provider’s standard protocol.

In order to investigate the presence of *Ace-1* duplication, purified DNA amplified as above from 12 G119S heterozygote mosquitoes was cloned using the Fermentas CloneJET™ PCR Cloning Kit, with colonies screened for the presence of the inserted amplicon using the supplied pJET1.2 primers according to the manufacturer’s instructions. Bands of approximately 900 bp were regarded as clones likely to contain the *Ace-1* insertion haplotype and sequenced (by Macrogen).

CodonCode Aligner version 3.7.1 was used to align DNA sequences with a consensus sequence from a resistant strain from multiple homozygous resistant individuals collected in Accra by S. N. Mitchell in 2008 [unpublished data] used as a reference. For the cloned samples, a BLAST search against *An. gambiae* PEST strain was performed to ascertain that no remnant of plasmid was present following. Forward and reverse sequences from each sample were arranged and checked for congruence and variants identified. MEGA 5 [30] was used to build a maximum likelihood tree from the aligned sequences following equalization of length. Sequences have GenBank accession numbers: KF771240- KF771247.

#### Data analysis

Owing to the different types of data present, for example those specific to single sites, those across all sites, and in some cases pairwise comparisons between sites, several different types of analysis were performed. Chi-square tests were used to analyse the association between molecular form and phenotypic resistance and between *Ace-1* genotype and resistance phenotype and were calculated, along with odds ratios where appropriate, using the Poptools version 3.1 add-in [31] for Microsoft Excel. The Hardy-Weinberg equation was used to calculate the expected genotype frequency in a panmictic population and tested using chi-square. Simple and partial Mantel tests were performed to assess the possible relation between matrices using ZT [32]. This analysis was performed using pair-wise differences in proportion of S-form, *Ace-1* 119S frequency, mortality rate, geographic distance, and also habitat and ecozones. The latter two were scored as 1 or 0 dummy variables based on whether sample sites originated from the same habitat class or ecozone. Partial Mantel tests were also performed holding the 119S frequency constant. Permutation p-values (100,000 permutations) were used to determine the significance of correlations. Binary logistic regression in SPSS version 20 was used to generate a model for the dichotomous categorical outcomes of the phenotype resistance (live and dead). *Anopheles coluzzii* and 119G homozygote were used as the reference categories for molecular form and genotype, respectively, to calculate odds ratios with 95% confidence interval.

## Results

**Figure 2:**
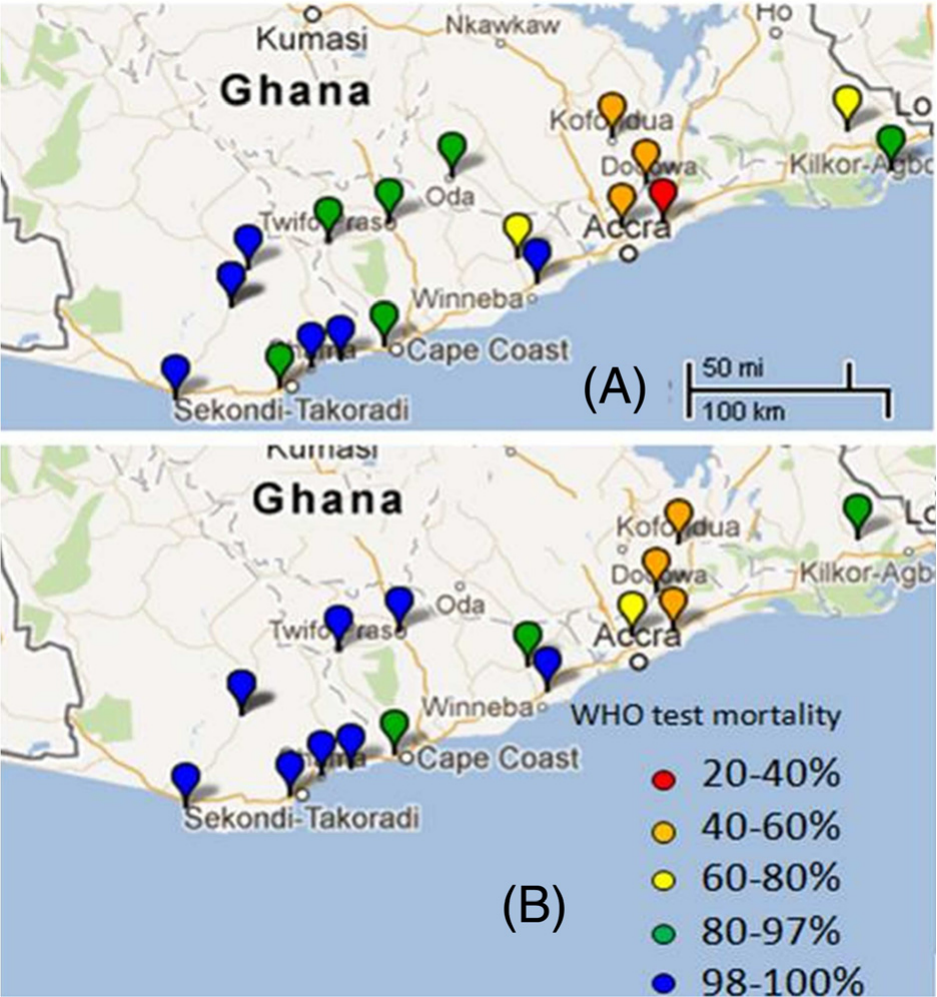
Prevalence of carbamate (A) and organophosphate (B) resistance assessed by WHO bioassays.

**Figure 3:**
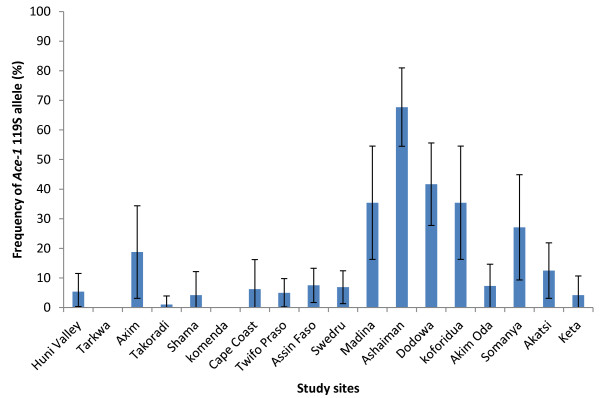
**Frequency of the *Ace-1 *** **119S allele across study sites (+/- 95% confidence intervals).**

**Figure 4:**
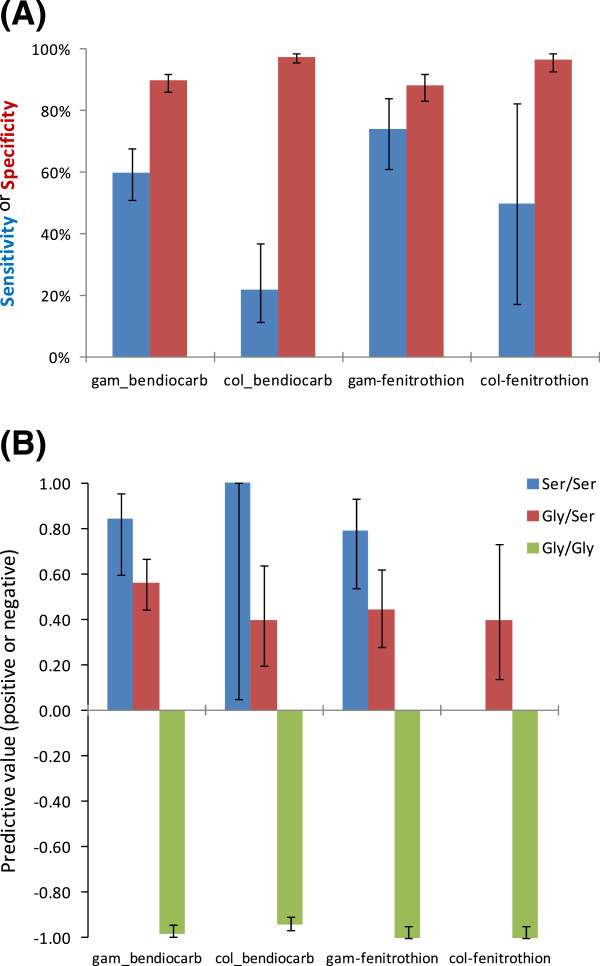
**Diagnostic value of *Ace-1 *alleles (A) and genotypes (B) for detection of resistance in *An. gambiae s.s. *(gam) and *An. coluzzii *(col). (A)** Bars show estimates of sensitivity (blue) and specificity (red) with 95% confidence limits. **(B)** Bars show positive predictive values for genotypes possessing resistance-associated alleles and negative predictive values for those possessing only wild type alleles; each with 95% confidence intervals.

A total of 3,946 adult female mosquitoes from 18 study sites were bioassayed: 17 with bendiocarb and 14 with fenitrothion (Figure 2). Mortality rates were highly variable ranging from 25-100% for bendiocarb and from 50-100% for fenitrothion, the latter being significantly higher across sites in which both insecticides were tested (Wilcoxon test, P = 0.008). However, the mortality rates of the two insecticides were strongly correlated (r = 0.93, P = 0.001). Using diagnostic PCRs, species identity for all of the 902 mosquitoes from the bioassays examined proved to be the *An. gambiae s.s.*species pair, with a subsample of 788 identified further as 53.3% *An. gambiae s.s.* and 46.6% *An. coluzzii* (Table 1) and only one interspecific (i.e. M/S) hybrid. *Ace-1* G119S genotype was characterized in the 788 *An. coluzzii* and *An. gambiae s.s*; of these, 601 (76.3%) were homozygous GG (wild-type), 149 (18.8%) heterozygous GS and 39 (4.9%) homozygous SS. The 119S allele was found in 16 of the 18 study sites, often at low frequency (<10% in ten sites) but with high frequencies in collections from Ashaiman (68%), Dodowa (42%), Koforidua (35%) and Madina (Accra) (35%) in the centre of the sampling range (Figure 3). The overall *Ace-1* 119S mutation frequency was 14.3% (95% CI: 11.9-16.8%), but with a significantly higher frequency (χ^2^ = 92.9, P = 5 x 10^-22^) in *An. gambiae s.s* (23.1%, 95% CI: 20.3-26.0%) than in *An. coluzzii* (4.4%, 95% CI: 2.9-5.8%). G119S was strongly associated with both bendiocarb and fenitrothion resistance with odds ratios (OR) of 15.7 (95% CI: 10.5-23.6) and 28.8 (95% CI: 15.4-53.9) respectively (see Additional file 2). Using samples pooled across study sites, G119S genotypic frequencies within *An. coluzzii* showed no deviations from Hardy-Weinberg equilibrium (HWE χ^2^ = 0.71, df = 1, P = 0.14). Deviation from HWE was found in *An. gambiae s.s.* (χ^2^ = 18.36 df = 1, P = 1.8 x 10^-5^) and was attributable to an excess of homozygotes. However, closer examination of individual sample site data revealed that this resulted primarily from an exceptionally high frequency of SS homozygotes in Ashaiman (Table 1), exclusion of which returned the data overall to HWE (χ^2^ = 0.37 df = 1, P = 0.54). A binary logistic regression analysis was performed to investigate molecular form and *Ace-1* genotype as predictors of phenotypic resistance for bendiocarb. Due to the detection of only a single homozygous-serine genotype in *An. coluzzii*, the analysis was performed using heterozygous and homozygote glycine individuals. The analysis was not performed for fenitrothion because no homozygote GG individuals survived the bioassay. G119S genotype and the genotype x species interaction term both made a significant contribution to the model (Additional file 3). Consistent with this interaction, the diagnostic capacity of *Ace-1* as a resistance marker differed between *An. coluzzii* and *An. gambiae s.s* for bendiocarb, with high specificity for both species but markedly lower sensitivity for *An. coluzzii* than *An. gambiae s.s.* (Figure 4A). Smaller sample sizes for fenitrothion survivors reduced power to evaluate possible differences in sensitivity between species or insecticides, but notably, specificity was high for *An. coluzzii* and *An. gambiae s.s.* with both insecticides, and generally much higher than specificity. In other words, absence of a serine allele at the 119 codon of *Ace-1* gives a very strong indication of carbamate and organophosphate susceptibility, but possession of a serine allele is only a moderately good predictor of resistance, and quite poor in *An. coluzzii*. By contrast, homozygosity of serine alleles provided high positive predictive value (Figure 4B).

**Table 1:** ***Ace-1 *** **G119S genotype distribution from the collection sites**

	***An. coluzzii***			***An. gambiae s.s.***		
	**SS**	**GS**	**GG**	**SS**	**GS**	**GG**
Huni Valley	0	0	29	1	4	22
Tarkwa	0	0	46	0	0	2
Axim	0	0	1	0	9	14
Takoradi	0	1	47	0	0	0
Shama	0	1	22	0	1	0
Komenda	0	0	22	0	0	2
Cape Coast	0	3	21	0	0	0
Twifo Praso	0	0	30	1	6	43
Assin Faso	0	2	23	0	10	45
Swedru	0	0	24	0	11	44
Madina	0	0	1	4	9	10
Ashaiman	1	13	7	23	4	0
Dodowa	0	2	1	3	32	10
Koforidua	0	1	0	3	10	10
Akim Oda	0	1	24	0	6	17
Somanya	0	2	0	3	5	14
Akatsi	0	1	5	0	11	31
Keta	0	3	33	0	0	0
Total	1	30	336	38	118	264

A simple Mantel test was performed to determine the correlation of bendiocarb mortality across populations with different explanatory variables. *Ace-1* 119S frequency was found to be very strongly correlated, while relative frequency of *An. gambiae s.s.* (i.e. S-form), distance and ecozones correlated marginally with mortality (Table 2). A partial Mantel test was then performed holding the most strongly correlated variable, A*ce- 119S* frequency, constant to evaluate the influence of other variables: only habitat showed a correlation close to significance. This test was not performed for fenitrothion due to smaller sample size.

**Table 2:** Correlations between bendiocarb mortality and explanatory variables

	**Simple mantel test**		**Partial mantel test**	
**Variable**	**r**	**P-value**	**r**	**P-value**
*Ace-1* 119S frequency	0.677	0.0002		
Species	0.147	0.059	-0.010	0.513
Distance	0.190	0.056	0.110	0.121
Habitat	-0.012	0.563	-0.123	0.043
Ecozone	0.149	0.068	0.080	0.204

### *Characterization of* Ace-1 *sequence*

**Figure 5:**
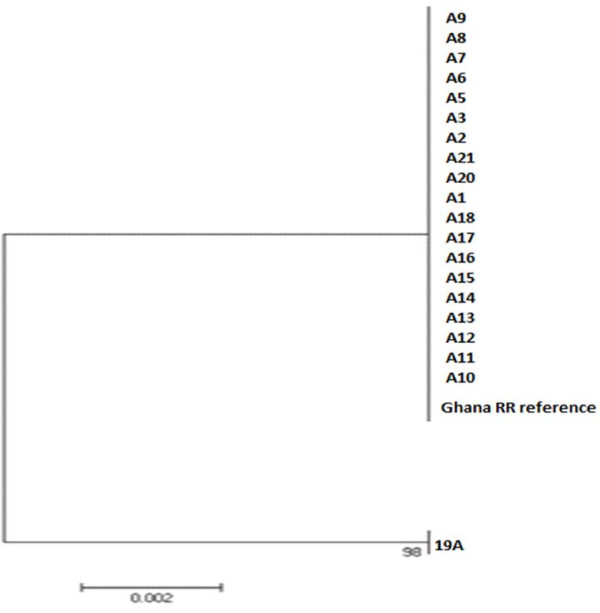
**Maximum likelihood tree of *Ace-1 *variation from direct sequencing.** All individuals are homozygous serine apart from 19A which is a homozygous glycine individual (S-form). A15 is the only *An. coluzzii* homozygous resistant individual (no others detected in whole dataset).

Twenty individuals homozygous for the 119S allele (19 *An. gambiae s.s*. and the single *An. coluzzii* serine homozygote found) from six study sites and one 119 GG individual were analysed to determine polymorphism within 119S alleles to investigate mutational origins. All the sequences bearing the *Ace-1* 119S allele were identical regardless of their molecular form or geographical origin, and all were identical to a consensus reference sequence from previously characterized samples collected in 2008 from Madina (Figure 5). By contrast, excluding the G119S substitution, 13 polymorphic positions were detected between the serine allele and the single homozygous 119G individual.

**Figure 6:**
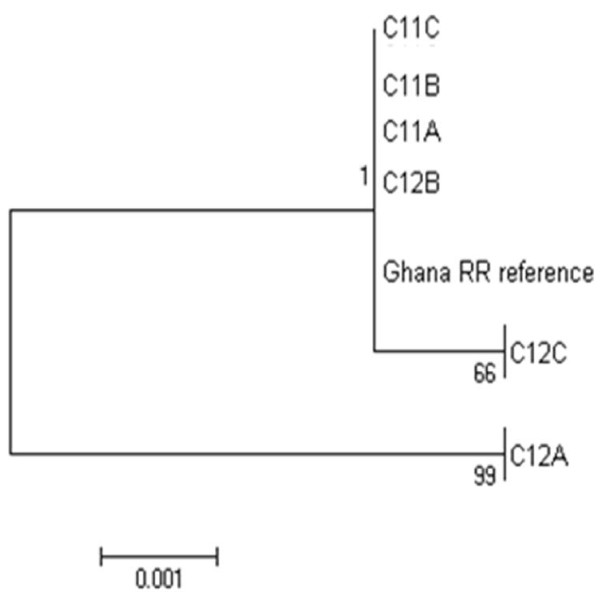
***Ace-1 *alleles from cloned haplotypes.** C11 typed as homozygous resistant in a Taqman assay, C12 typed as heterozygous.

Sequencing of cloned alleles was performed to detect the possible presence of *Ace-1* duplication(s), which would be evident if more than two alleles are present in an individual. As a result of low cloning efficiency only two individuals (both *An. coluzzii*; one homozygous serine and one heterozygote from Taqman genotyping) provided the minimum of three successfully cloned haplotypes each, necessaryto allow detection of duplication. The Taqman SS individual contained only a single allele, identical to the reference sequence (Figure 6), confirming direct sequencing results (Figure 5). However, the Taqman GS-genotyped individual contained three different serine alleles (C12A, C12B and C12C) (Figure 6). C12B and C12C differ by one base, which might be attributed to either PCR error or genuinely alternate alleles. However, ten variants were found between C12B and C12A (eight in intron 5 and two in exon 6 from Vectorbase). Owing to the expectation of a glycine allele in the sequences, presumably missing as a result of sampling error with only three clones, detection of two highly different serine haplotypes provides a strong indication of duplication (Additional file 4).

## Discussion

### Distribution of resistance

Insecticide resistance can be very heterogeneous even over relatively small distances [33]; a phenomenon clearly evident in *An. gambiae* in southern Ghana for both fenitrothion and bendiocarb, susceptibility to which varied markedly even within regions. Indeed, geographical proximity between sample sites was a poor general predictor of resistance. Despite this variability, some geographical patterning of resistance can be seen, with a tendency for higher levels in the greater Accra area, which decreases toward the west and east. This might indicate that resistance originated in or near Greater Accra and has spread to other parts of the country. Barring long-range transport of resistant mosquitoes this would suggest a unique local origin either by mutation or from standing variation. Another possible explanation could be that resistance might have originated elsewhere but increased to higher levels in the Greater Accra area due to higher selection pressure. Although habitat did not correlate well with phenotypic mortality, only broad characterization of selective environment was attempted. Use of carbamate and organophosphate insecticides for malaria control has been very limited to date in Ghana, thus, the primary source of selection must be from agricultural usage, for which data is difficult to obtain. Nevertheless a high prevalence of resistance in Ashaiman, a locale characterized by large-scale cultivation of rice and vegetables, which relies extensively on pesticide application, is consistent with agriculture-driven selection. Resistance levels in the Accra suburb of Madina, and also Koforidua, have a less clear agricultural link, but might be attributed to pesticide use across a myriad of small-scale vegetable farms. Previous work by Tallaki [34] and Klinkenberg *et al*. [35] in West Africa associated a high level of insecticide resistance in malaria vectors to rapid expansion in urban agriculture wherein insecticide deployment is usually intensive and under-regulated [36].

In the western sample sites, relatively low levels of resistance may indicate different selection pressure originating from a predominance of “dry cultivation” such as large scale cocoa and rubber plantations. Hunt *et al.*[20] reported resistance to bendiocarb in *An. gambiae s.s.* from Tarkwa (Western region) whereas complete susceptibility was observed in the present study. The marked disparity in resistance status could be attributable to different mosquito collection points because Hunt *et al.*[20], sampled larvae directly in the mining centre which is isolated from the main town where the collections from the present study were performed. This further suggests a fine geographical grain across which resistance levels can vary.

### Ace-1 *mutation*

The *Ace-1* G119S mutation was found to be very strongly associated with phenotypic resistance to both insecticides. Homozygous wild type (GG) individuals very rarely survived exposure to either insecticide, whereas homozygous serine individuals were very likely to survive, yielding high negative and positive predictive value as a diagnostic, respectively. The predictive value for heterozygotes was much more moderate, and allelic sensitivity was much poorer than specificity. *Ace-1* 119S frequency was highly variable across southern Ghana, being absent from some samples but present in Ashaiman at the highest frequency yet recorded, far exceeding a report of 50% from Côte d’lvoire [15]. Both the logistic regression and Mantel test analyses indicated that *Ace-1* 119S frequency acts as a strong independent predictor of mortality to bendiocarb. Of other variables investigated, habitat, ecological zone and distance were marginally or non-significantly associated with resistance suggesting that if they exert an influence, it is in a weaker or more complex way than could be identified from the present dataset. Both Asidi *et al.*[37] and Djogbenou *et al.*[14] documented that G119S had a greater impact on carbamate than organophosphate resistance in *An. gambiae*. The present results are consistent with these findings but resistance levels were highly correlated across populations and, as also reported by Edi *et al.*[16], G119S was significantly associated with both fenitrothion and bendiocarb resistance.

Frequencies of the G119S mutation linked to an insensitive acetylcholinesterse phenotype have been reported from neighbouring Côte d’Ivoire [15] and Burkina Faso [38], but prior to the present study little information was available for Ghana. The *Ace-1* 119S mutation was detected in both *An. coluzzii* and *An. gambiae s.s.*, but was significantly more common in *An. gambiae s.s.* (23.5%) than in *An. coluzzii* (4.3%). A similar difference in frequencies between molecular forms (32% in *An. gambiae s.s.* and 3.6% in *An. coluzzii*) has been reported in Burkina Faso [38], but not in Côte d’Ivoire [15] where 119S *An. coluzzii* and *An. gambiae s.s.* frequencies are similar (31% and 35%, respectively). *Anopheles coluzzii* and *An. gambiae s.*s., which are sympatric throughout much of southern Ghana, also exhibited significantly different levels of resistance to both insecticides, with *An. gambiae s.s.* much more resistant. Interestingly however, species was not a significant predictor of resistance when *Ace-1* 119S genotype was included in models, suggesting that the primary cause of the difference relates to the target site polymorphism rather than wider genomic background in these samples. It has been suggested that the difference in 119S mutation frequency between forms may be due to discrepancies in the selection pressure they experience in their habitats [38]. Yet, given the typically greater propensity for *An. coluzzii* to occur in agricultural habitats [39-42] later occurrence of 119S in *An. coluzzii*, either by independent mutation or introgression might be a more likely explanation. Unfortunately, as reported previously for samples from other West African countries [43], sequence analysis of the resistant allele proved uninformative with respect to origin, owing to a complete lack of sequence variation. Unless there is very strong selection for the particular haplotype detected, this is more consistent with spread of a recent mutation than multiple origins or selection from standing variation. There is one intriguing additional possible explanation for the difference in 119S frequency between *An. coluzzii* and *An. gambiae s.s*. Although both insecticides appear to be effective in killing the *Ace-1* homozygous wild-type individuals, a significant interaction was found between *Ace-1* genotype and species, such that heterozygous individuals are more resistant in *An. gambiae s.s* than *An. coluzzii*. The mechanistic reason for this is unclear but might involve the presence of additional, non-target site mechanisms or perhaps differences in the frequency or nature of *Ace-1* duplication between molecular forms.

### Ace-1 *genotype distributions and duplication*

With the exception of Ashaiman where 85% of *An. gambiae s.s* were found to be 119S homozygotes, very low frequencies of 119S homozygotes were found, with a complete absence in 11 localities. This is consistent with previous work in Côte d’Ivoire [15], and a relative paucity of *Ace-1* 119S homozygotes might be expected for two reasons. Extensive studies of the functional consequences of the G119S mutation in *Culex pipiens* has revealed that the mutation leads to a reduction in the normal function of acetylcholinesterase, lower male reproductive success, and lower female survivorship during overwintering [44-47]. Consequently, in the absence of selection pressure from carbamate and organophosphate exposure, G119S homozygotes are not detected after few generations [47], which has important implications for insecticide resistance management [2]. Djogbénou *et al.*[48] also documented higher pupal mortality in homozygous 119S individuals than those homozygous for 119G in *An. gambiae s.s.* Such previous findings make the detection of an exceptionally high frequency of serine homozygotes in Ashaiman particularly striking.

The other explanation for a paucity of 119S homozygotes is the presence of *Ace-1* copy number variation. Albeit from a single sample in this study, it confirmed that *An. gambiae* in southern Ghana can possess three distinct *Ace-1* alleles, as previously documented for samples from Côte d’Ivoire and Burkina Faso [43], which indicates the presence of duplication. However, in contrast to these previous findings [43], the sequenced individual specimen possessed at least two distinct resistant alleles and presumably one (undetected) susceptible allele. Previous studies in *Cx. pipiens*, have revealed that duplication is associated with a lower fitness cost than the single-copy resistance homozygote and might be selected as a compensatory mechanism for the fitness cost of the homozygous 119S allele, eventually resulting in the fixation of heterozygosity [44]. Owing to very serious consequences for insecticide resistance management if *Ace-1* 119S can be sustained with little fitness cost in the absence of insecticide, investigation of *Ace-1* duplication in *An. gambiae* should represent a high priority for future research.

## Conclusions

The present study revealed high variability in carbamate and organophosphate resistance across southern Ghana, though with levels correlated across insecticides and generally much higher prevalence in S molecular forms. The G119S mutation was found to be by far the strongest predictor of resistance to both insecticides and at present is the best molecular resistance diagnostic available for any insecticide in *An. gambiae*, with high sensitivity (for absence of resistance) and good positive predictive value for serine homozygotes, which are currently quite rare. Whilst absence of the 119S allele strongly predicted susceptibility, presence was a less reliable indicator of survival, translating into only moderate specificity and with a difference apparent in diagnostic capacity between molecular forms. This strongly implicates involvement of one or more additional mechanisms, which add an increased layer of resistance, a candidate for which might be *Ace-1* amplification, which was found to be present. By evaluating the diagnostic capabilities of the *Ace-1* 119 locus the findings of the present study can help understanding and prediction of the spread of carbamate and organosphosphate resistance in *An. gambiae s.s.* populations, and to aid rational planning of insecticide deployment in Ghana and more widely in West Africa.

## Competing interests

The authors declare that they have no competing interests.

## Authors’ contributions

DW and AEY conceived of the study, and all authors participated in the design. JE performed field and molecular laboratory work, coordinated by AEY and DW. JE and DW drafted the manuscript. DW and JE analysed the data. All authors read and approved the final manuscript.

## Supplementary Material

Additional file 1 Study site classification. Click here for file

Additional file 2 Genotypic and allelic frequencies and association tests. Click here for file

Additional file 3 Logistic regression model for bendiocarb resistance. Click here for file

Additional file 4 **Alignment of cloned *Ace-1 *** **sequences.** Click here for file
