# Supplementary material for: Acetylcholinesterase (Ace-1) target site mutation 119S is strongly diagnostic of carbamate and organophosphate resistance in Anopheles gambiae s.s. and Anopheles coluzzii across southern Ghana
Source: Malar J. 2013 Nov 9;12:404. doi: 10.1186/1475-2875-12-404 (PMC3842805; doi:10.1186/1475-2875-12-404)
Supplement: Additional file 1 — Study site classification. [file 1475-2875-12-404-S1.pdf]

Additional file 1. Study site classification

Location	ID	Latitude	Longitude	Sampling date	Settlement	Ecological zone (Ecozone)	Agricultural activity
Huni Valley	1	5.473840N	1.915080W	07/06/2011	Rural	Rainforest	Dry cultivation
Tarkwa	2	5.299550N	01.993060W	06/06/2011	Urban	Rainforest	Dry cultivation
Axim	3	4.864470N	02.242630W	06/06/2011	Peri-urban	Rainforest	Dry cultivation
Twifo Praso	7	5.608580N	01.549260W	21/06/2011	Peri-urban	Deciduous forest	Dry cultivation
Assin Foso	9	5.689110N	01.271450W	21/06/2011	Peri-urban	Deciduous forest	Dry cultivation
Akim Oda	11	5.913140N	00.987610W	30/06/2011	Peri-urban	Deciduous forest	Semi-irrigation
Koforidua	15	6.094490N	0.260930W	03/06/2011	Urban	Deciduous forest	Semi-irrigation
Somanya	16	6.103550N	00.010570W	20/06/2011	Peri-urban	Deciduous forest	Dry cultivation
Akatsi	17	6.13396N	00.80092E	27/08/2011	Peri-urban	Guinea savanna	Semi-irrigation
Takoradi	4	4.912170N	1.773970 W	16/06/2011	Urban	Coastal savanna	Semi-irrigation
Shama	5	5.011760N	05.050180W	19/06/2011	Peri-urban	Coastal savanna	Semi-irrigation
Komenda	6	5.050180N	05.050180W	19/06/2011	Peri-urban	Coastal savanna	Semi-irrigation
Swedru	10	5.529440N	0.696980W	20/07/2011	Urban	Coastal savanna	Dry cultivation
Madina	12	5.668490N	0.219280W	29/05/2011	Urban	Coastal savanna	Semi-irrigation
Dodowa	14	5.880970N	0.107880W	20/05/2011	Peri-urban	Coastal savanna	Dry cultivation
Cape Coast	8	5.108280N	1.296770W	01/08/2011	Urban	Mangrove strand	Semi-irrigation
Ashaiman	13	5.693080N	0.033230W	19/05/2011	Peri-urban	Mangrove strand	Semi-irrigation
Keta	18	5.939940N	0.996250E	26/08/2011	Peri-urban	Mangrove strand	Irrigation

Irrigation refers to “permanent water” created for rice farms. Semi-irrigation refers to temporary water created for vegetable farms. Dry cultivation refers to cash crop plantations (oil palm, rubber, and mango) that create little or no water containment.
