# Supplementary material for: Acetylcholinesterase (Ace-1) target site mutation 119S is strongly diagnostic of carbamate and organophosphate resistance in Anopheles gambiae s.s. and Anopheles coluzzii across southern Ghana
Source: Malar J. 2013 Nov 9;12:404. doi: 10.1186/1475-2875-12-404 (PMC3842805; doi:10.1186/1475-2875-12-404)
Supplement: Additional file 2 — Genotypic and allelic frequencies and association tests. [file 1475-2875-12-404-S2.pdf]

Additional file 2: Genotypic and allelic frequencies and association tests.

A: bendiocarb; *An. gambiae s.s.*

Locality	Dead			Live			Sample size
	SS	GS	GG	SS	GS	GG	
Huni Valley	1	3	22	0	1	0	27
Tarkwa	0	0	1	0	0	0	1
Axim	0	8	14	0	1	0	23
Takoradi	0	0	0	0	0	0	0
Shama	0	1	0	0	0	0	1
Komenda	0	0	2	0	0	0	2
Cape Coast	0	0	0	0	0	0	0
Twifo Praso	0	4	27	0	0	1	32
Assin Faso	0	6	31	0	2	1	40
Swedru	0	2	11	0	4	0	17
Madina	0	1	10	4	8	0	23
Ashaiman	2	1	0	8	1	0	12
Dodowa	0	4	7	1	12	0	24
Koforidua	0	2	9	3	8	1	23
Akim Oda	0	1	17	0	5	0	23
Somanya	0	0	0	0	0	0	0
Akatsi	0	3	15	0	4	0	22
Keta	0	0	0	0	0	0	0
Total	3	36	166	16	46	3	270

Genotypic chi-square test for bendiocarb *An. gambiae s.s.*

	SS	GS	GG
**Dead**	**3**	**36**	**166**
**Live**	**16**	**46**	**3**

$X^2 = 129.57$, df = 2, P < 0.0001

Allelic chi-square test for bendiocarb *An. gambiae s.s.*

	S	G
**Dead**	**42**	**368**
**Live**	**78**	**52**

$X^2 = 141.38$, df = 1, P < 0.0001

Odds ratio (OR) = 13.14 (95% C.I. 8.18-21.21)

B: bendiocarb; *An. coluzzii*

Locality	Dead			Live			Sample size
	SS	GS	SS	SS	GS	GG	
Huni Valley	0	0	29	0	0	0	29
Tarkwa	0	0	23	0	0	0	23
Axim	0	0	1	0	0	0	1
Takoradi	0	1	20	0	0	3	24
Shama	0	1	22	0	0	0	23
Komenda	0	0	21	0	0	1	22
Cape Coast	0	0	15	0	3	6	24
Twifo Praso	0	0	22	0	0	2	24
Assin Faso	0	2	14	0	0	0	16
Swedru	0	0	7	0	0	0	7
Madina	0	0	1	0	0	0	1
Ashiaman	0	6	3	1	2	0	12
Dodowa	0	0	0	0	0	0	0
Koforidua	0	1	0	0	0	0	1
Akim Oda	0	1	23	0	0	1	25
Akatsi	0	0	2	0	0	0	2
Keta	0	0	32	0	3	1	36
**Total**	0	12	235	1	8	14	270

Genotypic chi-square test for bendiocarb *An. coluzzii*

	SS	GS	GG
**Dead**	**0**	**12**	**235**
**Live**	**1**	**8**	**14**

$X^2 = 38.86$, df = 2, P < 0.0001

Allelic chi-square test for bendiocarb *An. coluzzii*

	S	G
**Dead**	**12**	**482**
**Live**	**10**	**36**

$X^2 = 35.36$, df =1, P < 0.0001

OR (95% C.I.)= 11.16 (4.51-27.58)

C: fenitrothion; *An. gambiae s.s.*

**Locality**	**Dead**			**Live**			**Sample size**
	SS	GS	GG	SS	GS	GG	
Tarkwa	0	0	1	0	0	0	1
Twifo Praso	1	2	15	0	0	0	18
Assin Faso	0	2	13	0	0	0	15
Swedru	0	2	33	0	3	0	38
Ashiaman	0	2	0	13	0	0	15
Dodowa	0	3	3	2	13	0	21
Somanya	3	5	14	0	0	0	22
Akatsi	0	4	16	0	0	0	20
Total	4	20	95	15	16	0	150

Genotypic chi-square test for fenitrothion *An. gambiae s.s.*

	**SS**	**GS**	**GG**
**Dead**	**4**	**20**	**95**
**Live**	**15**	**16**	**0**

X^2 = 76.52, df = 2, P < 0.0001

Allelic chi-square test for fenitrothion *An. gambiae s.s.*

	**S**	**G**
**Dead**	**28**	**210**
**Live**	**46**	**16**

X^2 = 103.16, df = 1, P < 0.0001

OR (95% C.I.) = 21.56 (10.79-43.08)

D: fenitrothion; *An. coluzzii*

Locality	Dead			Live			Sample size
	SS	GS	GG	SS	GS	GG	
Tarkwa	0	0	23	0	0	0	23
Takoradi	0	0	24	0	0	0	24
Twifo Praso	0	0	6	0	0	0	6
Assin Faso	0	0	9	0	0	0	9
Swedru	0	0	17	0	0	0	17
Ashiaman	0	2	4	0	3	0	9
Dodowa	0	1	1	0	1	0	3
Somanya	0	2	0	0	0	0	2
Akatsi	0	1	3	0	0	0	4
Total	0	6	87	0	4	0	97

Genotypic test for fenitrothion *An. coluzzii*

	**SS**	**GS**	**GG**
**Dead**	**0**	**6**	**87**
**Live**	**0**	**4**	**0**

P < 0.0001, fisher exact test used owing to low expected frequencies (http://www.vassarstats.net/fisher2x3.html)

Allelic chi-square test for fenitrothion *An. coluzzii*

	**S**	**G**
**Dead**	**6**	**180**
**Live**	**4**	**5**

X^2 = 22.10, df = 1, P < 0.0001

OR (95% C.I.) = 30.0 (6.02-149.63)
