# Supplementary material for: Acetylcholinesterase (Ace-1) target site mutation 119S is strongly diagnostic of carbamate and organophosphate resistance in Anopheles gambiae s.s. and Anopheles coluzzii across southern Ghana
Source: Malar J. 2013 Nov 9;12:404. doi: 10.1186/1475-2875-12-404 (PMC3842805; doi:10.1186/1475-2875-12-404)
Supplement: Additional file 3 — Logistic regression model for bendiocarb resistance. [file 1475-2875-12-404-S3.pdf]

Additional file 3: Logistic regression model for bendiocarb resistance

	B	S.E.	Wald	df	Sig.	Exp(B)	95% C.I.for EXP(B)	
							Lower	Upper
Species	-.651	.508	1.642	1	.200	.522	.193	1.412
Genotype	-4.258	.624	46.634	1	.000	.014	.004	.048
Species x Genotype	1.843	.820	5.050	1	.025	6.318	1.266	31.538
Constant	.245	.223	1.213	1	.271	1.278		
