# Supplementary material for: Acetylcholinesterase (Ace-1) target site mutation 119S is strongly diagnostic of carbamate and organophosphate resistance in Anopheles gambiae s.s. and Anopheles coluzzii across southern Ghana
Source: Malar J. 2013 Nov 9;12:404. doi: 10.1186/1475-2875-12-404 (PMC3842805; doi:10.1186/1475-2875-12-404)
Supplement: Additional file 4 — Alignment of cloned Ace-1 sequences. [file 1475-2875-12-404-S4.pdf]

Additional file 4: Alignment of cloned *Ace-1* sequences from two G/S heterozygote (C11 and C12; diagnosed by TaqMan) and from direct sequencing of Taqman SS homozygote samples from the present study and from a previous collection from Madina (Accra) in 2008.

```
C11A                            GACGCGGTCTGACGAGGCGCGAGTCAAACTCGGGTAAGTACGCGATTGGA 50
C11B                            GACGCGGTCTGACGAGGCGCGAGTCAAACTCGGGTAAGTACGCGATTGGA 50
C11C                            GACGCGGTCTGACGAGGCGCGAGTCAAACTCGGGTAAGTACGCGATTGGA 50
C12A                            GACGCGGTCTGACGAGGCGCGAGTCAAACTCGGGTAAGTACGCGATTGGA 50
C12B                            GACGCGGTCTGACGAGGCGCGAGTCAAACTCGGGTAAGTACGCGATTGGA 50
C12C                            GACGCGGTCTGACGAGGCGCGAGTCAAACTCGGGTAAGTACGCGATTGGA 50
Madina_2008_Ser_homozygote      GACGCGGTCTGACGAGGCGCGAGTCAAACTCGGGTAAGTACGCGATTGGA 50
Direct_seq_Ser_homozygotes      GACGCGGTCTGACGAGGCGCGAGTCAAACTCGGGTAAGTACGCGATTGGA 50
                                **************************************************

C11A                            AGTGGGGGGACGTTTACCCTACCGTGTACTACAACGCACTTTACCCCCAC 100
C11B                            AGTGGGGGGACGTTTACCCTACCGTGTACTACAACGCACTTTACCCCCAC 100
C11C                            AGTGGGGGGACGTTTACCCTACCGTGTACTACAACGCACTTTACCCCCAC 100
C12A                            AGTGGGGGGACGTTTACCCTACCGTGTACTACAACGCACTTTACCCCCAC 100
C12B                            AGTGGGGGGACGTTTACCCTACCGTGTACTACAACGCACTTTACCCCCAC 100
C12C                            AGTGGGGGGACGTTTACCCTACCGTGTACTACAACGCACTTTACCCCCAC 100
Madina_2008_Ser_homozygote      AGTGGGGGGACGTTTACCCTACCGTGTACTACAACGCACTTTACCCCCAC 100
Direct_seq_Ser_homozygotes      AGTGGGGGGACGTTTACCCTACCGTGTACTACAACGCACTTTACCCCCAC 100
                                **************************************************

C11A                            GCACACGCACCGGCAGACGCGAACGACAACGATCCGCTGGTGGTCAACAC 150
C11B                            GCACACGCACCGGCAGACGCGAACGACAACGATCCGCTGGTGGTCAACAC 150
C11C                            GCACACGCACCGGCAGACGCGAACGACAACGATCCGCTGGTGGTCAACAC 150
C12A                            GCACACGCACCGGCAGACGCGAACGACAACGATCCGCTGGTGGTCAACAC 150
C12B                            GCACACGCACCGGCAGACGCGAACGACAACGATCCGCTGGTGGTCAACAC 150
C12C                            GCACACGCACCGGCAGACGCGAACGACAACGATCCGCTGGTGGTCAACAC 150
Madina_2008_Ser_homozygote      GCACACGCACCGGCAGACGCGAACGACAACGATCCGCTGGTGGTCAACAC 150
Direct_seq_Ser_homozygotes      GCACACGCACCGGCAGACGCGAACGACAACGATCCGCTGGTGGTCAACAC 150
                                **************************************************

C11A                            GGATAAGGGGCGCATCCGCGGCATTACGGTCGATGCGCCCAGCGGCAAGA 200
C11B                            GGATAAGGGGCGCATCCGCGGCATTACGGTCGATGCGCCCAGCGGCAAGA 200
C11C                            GGATAAGGGGCGCATCCGCGGCATTACGGTCGATGCGCCCAGCGGCAAGA 200
C12A                            GGATAAGGGGCGCATCCGCGGCATTACGGTCGATGCGCCCAGCGGCAAGA 200
C12B                            GGATAAGGGGCGCATCCGCGGCATTACGGTCGATGCGCCCAGCGGCAAGA 200
C12C                            GGATAAGGGGCGCATCCGCGGCATTACGGTCGATGCGCCCAGCGGCAAGA 200
Madina_2008_Ser_homozygote      GGATAAGGGGCGCATCCGCGGCATTACGGTCGATGCGCCCAGCGGCAAGA 200
Direct_seq_Ser_homozygotes      GGATAAGGGGCGCATCCGCGGCATTACGGTCGATGCGCCCAGCGGCAAGA 200
                                **************************************************

C11A                            AGGTGGACGTGTGGCTCGGCATTCCCTACGCCCAGCCGCCGGTCGGGCCG 250
C11B                            AGGTGGACGTGTGGCTCGGCATTCCCTACGCCCAGCCGCCGGTCGGGCCG 250
C11C                            AGGTGGACGTGTGGCTCGGCATTCCCTACGCCCAGCCGCCGGTCGGGCCG 250
C12A                            AGGTGGACGTGTGGCTCGGCATTCCCTACGCCCAGCCGCCGGTCGGGCCG 250
C12B                            AGGTGGACGTGTGGCTCGGCATTCCCTACGCCCAGCCGCCGGTCGGGCCG 250
C12C                            AGGTGGACGTGTGGCTCGGCATTCCCTACGCCCAGCCGCCGGTCGGGCCG 250
Madina_2008_Ser_homozygote      AGGTGGACGTGTGGCTCGGCATTCCCTACGCCCAGCCGCCGGTCGGGCCG 250
Direct_seq_Ser_homozygotes      AGGTGGACGTGTGGCTCGGCATTCCCTACGCCCAGCCGCCGGTCGGGCCG 250
                                **************************************************

C11A                            CTACGGTTCCGTCATCCGCGGCCGGCCGAAAAGTGGACCGGCGTGCTGAA 300
C11B                            CTACGGTTCCGTCATCCGCGGCCGGCCGAAAAGTGGACCGGCGTGCTGAA 300
C11C                            CTACGGTTCCGTCATCCGCGGCCGGCCGAAAAGTGGACCGGCGTGCTGAA 300
C12A                            CTACGGTTCCGTCATCCGCGGCCGGCCGAAAAGTGGACCGGCGTGCTGAA 300
C12B                            CTACGGTTCCGTCATCCGCGGCCGGCCGAAAAGTGGACCGGCGTGCTGAA 300
C12C                            CTACGGTTCCGTCATCCGCGGCCGGCCGAAAAGTGGACCGGCGTGCTGAA 300
Madina_2008_Ser_homozygote      CTACGGTTCCGTCATCCGCGGCCGGCCGAAAAGTGGACCGGCGTGCTGAA 300
Direct_seq_Ser_homozygotes      CTACGGTTCCGTCATCCGCGGCCGGCCGAAAAGTGGACCGGCGTGCTGAA 300
                                **************************************************

C11A                            CACGACCACACCGCCCAACAGCTGCGTGCAGATCGTGGACACCGTGTTCG 350
C11B                            CACGACCACACCGCCCAACAGCTGCGTGCAGATCGTGGACACCGTGTTCG 350
C11C                            CACGACCACACCGCCCAACAGCTGCGTGCAGATCGTGGACACCGTGTTCG 350
C12A                            CACGACCACACCGCCCAACAGCTGCGTGCAGATCGTGGACACCGTGTTCG 350
C12B                            CACGACCACACCGCCCAACAGCTGCGTGCAGATCGTGGACACCGTGTTCG 350
C12C                            CACGACCACACCGCCCAACAGCTGCGTGCAGGTCGTGGACACCGTGTTCG 350
Madina_2008_Ser_homozygote      CACGACCACACCGCCCAACAGCTGCGTGCAGATCGTGGACACCGTGTTCG 350
Direct_seq_Ser_homozygotes      CACGACCACACCGCCCAACAGCTGCGTGCAGATCGTGGACACCGTGTTCG 350
                                ******************************* ******************
```

```
C11A                            GCGACTTCCCGGGCGCGACCATGTGGAACCCGAACACGCCCCTGTCCGAG 400
C11B                            GCGACTTCCCGGGCGCGACCATGTGGAACCCGAACACGCCCCTGTCCGAG 400
C11C                            GCGACTTCCCGGGCGCGACCATGTGGAACCCGAACACGCCCCTGTCCGAG 400
C12A                            GCGACTTCCCGGGCGCGACCATGTGGAACCCGAACACGCCCCTGTCCGAG 400
C12B                            GCGACTTCCCGGGCGCGACCATGTGGAACCCGAACACGCCCCTGTCCGAG 400
C12C                            GCGACTTCCCGGGCGCGACCATGTGGAACCCGAACACGCCCCTGTCCGAG 400
Madina_2008_Ser_homozygote      GCGACTTCCCGGGCGCGACCATGTGGAACCCGAACACGCCCCTGTCCGAG 400
Direct_seq_Ser_homozygotes      GCGACTTCCCGGGCGCGACCATGTGGAACCCGAACACGCCCCTGTCCGAG 400
                                **************************************************

C11A                            GACTGTCTGTACATTAACGTGGTGGCACCGCGGCCCCGGCCCAAGAATGC 450
C11B                            GACTGTCTGTACATTAACGTGGTGGCACCGCGGCCCCGGCCCAAGAATGC 450
C11C                            GACTGTCTGTACATTAACGTGGTGGCACCGCGGCCCCGGCCCAAGAATGC 450
C12A                            GACTGTCTGTACATTAACGTGGTGGCACCGCGGCCCCGGCCCAAGAATGC 450
C12B                            GACTGTCTGTACATTAACGTGGTGGCACCGCGGCCCCGGCCCAAGAATGC 450
C12C                            GACTGTCTGTACATTAACGTGGTGGCACCGCGGCCCCGGCCCAAGAATGC 450
Madina_2008_Ser_homozygote      GACTGTCTGTACATTAACGTGGTGGCACCGCGGCCCCGGCCCAAGAATGC 450
Direct_seq_Ser_homozygotes      GACTGTCTGTACATTAACGTGGTGGCACCGCGGCCCCGGCCCAAGAATGC 450
                                **************************************************

C11A                            GGCCGTCATGCTGTGGATCTTCGGCGGCAGCTTCTACTCCGGCACCGCCA 500
C11B                            GGCCGTCATGCTGTGGATCTTCGGCGGCAGCTTCTACTCCGGCACCGCCA 500
C11C                            GGCCGTCATGCTGTGGATCTTCGGCGGCAGCTTCTACTCCGGCACCGCCA 500
C12A                            GGCCGTCATGCTGTGGATCTTCGGCGGCAGCTTCTACTCCGGCACCGCCA 500
C12B                            GGCCGTCATGCTGTGGATCTTCGGCGGCAGCTTCTACTCCGGCACCGCCA 500
C12C                            GGCCGTCATGCTGTGGATCTTCGGCGGCAGCTTCTACTCCGGCACCGCCA 500
Madina_2008_Ser_homozygote      GGCCGTCATGCTGTGGATCTTCGGCGGCAGCTTCTACTCCGGCACCGCCA 500
Direct_seq_Ser_homozygotes      GGCCGTCATGCTGTGGATCTTCGGCGGCAGCTTCTACTCCGGCACCGCCA 500
                                **************************************************

C11A                            CCCTGGACGTGTACGACCACCGGGCGCTTGCGTCGGAGGAGAACGTGATC 550
C11B                            CCCTGGACGTGTACGACCACCGGGCGCTTGCGTCGGAGGAGAACGTGATC 550
C11C                            CCCTGGACGTGTACGACCACCGGGCGCTTGCGTCGGAGGAGAACGTGATC 550
C12A                            CCCTGGACGTGTACGACCACCGGGCGCTTGCGTCGGAGGAGAACGTGATC 550
C12B                            CCCTGGACGTGTACGACCACCGGGCGCTTGCGTCGGAGGAGAACGTGATC 550
C12C                            CCCTGGACGTGTACGACCACCGGGCGCTTGCGTCGGAGGAGAACGTGATC 550
Madina_2008_Ser_homozygote      CCCTGGACGTGTACGACCACCGGGCGCTTGCGTCGGAGGAGAACGTGATC 550
Direct_seq_Ser_homozygotes      CCCTGGACGTGTACGACCACCGGGCGCTTGCGTCGGAGGAGAACGTGATC 550
                                **************************************************

C11A                            GTGGTGTCGCTGCAGTACCGCGTGGCCAGTCTGGGCTTCCTGTTTCTCGG 600
C11B                            GTGGTGTCGCTGCAGTACCGCGTGGCCAGTCTGGGCTTCCTGTTTCTCGG 600
C11C                            GTGGTGTCGCTGCAGTACCGCGTGGCCAGTCTGGGCTTCCTGTTTCTCGG 600
C12A                            GTGGTGTCGCTGCAGTACCGCGTGGCCAGTCTGGGCTTCCTGTTTCTCGG 600
C12B                            GTGGTGTCGCTGCAGTACCGCGTGGCCAGTCTGGGCTTCCTGTTTCTCGG 600
C12C                            GTGGTGTCGCTGCAGTACCGCGTGGCCAGTCTGGGCTTCCTGTTTCTCGG 600
Madina_2008_Ser_homozygote      GTGGTGTCGCTGCAGTACCGCGTGGCCAGTCTGGGCTTCCTGTTTCTCGG 600
Direct_seq_Ser_homozygotes      GTGGTGTCGCTGCAGTACCGCGTGGCCAGTCTGGGCTTCCTGTTTCTCGG 600
                                **************************************************

C11A                            CACCCCCGAAGCGCCGGGCAATGCGGGACTGTTCGATCAGAACCTTGCGC 650
C11B                            CACCCCCGAAGCGCCGGGCAATGCGGGACTGTTCGATCAGAACCTTGCGC 650
C11C                            CACCCCCGAAGCGCCGGGCAATGCGGGACTGTTCGATCAGAACCTTGCGC 650
C12A                            CACCCCCGAAGCGCCGGGCAATGCGGGACTGTTCGATCAGAACCTTGCGC 650
C12B                            CACCCCCGAAGCGCCGGGCAATGCGGGACTGTTCGATCAGAACCTTGCGC 650
C12C                            CACCCCCGAAGCGCCGGGCAATGCGGGACTGTTCGATCAGAACCTTGCGC 650
Madina_2008_Ser_homozygote      CACCCCCGAAGCGCCGGGCAATGCGGGACTGTTCGATCAGAACCTTGCGC 650
Direct_seq_Ser_homozygotes      CACCCCCGAAGCGCCGGGCAATGCGGGACTGTTCGATCAGAACCTTGCGC 650
                                **************************************************

C11A                            TACGGTAGGTGTCTTTGCGTGTGTGTCTGTAGTTATAGTATTCTAACGAG 700
C11B                            TACGGTAGGTGTCTTTGCGTGTGTGTCTGTAGTTATAGTATTCTAACGAG 700
C11C                            TACGGTAGGTGTCTTTGCGTGTGTGTCTGTAGTTATAGTATTCTAACGAG 700
C12A                            TACGGTAGGTGTCTTTGCATGTGTGAGTGAGGCTTTAGTATTCTAACGAG 700
C12B                            TACGGTAGGTGTCTTTGCGTGTGTGTCTGTAGTTATAGTATTCTAACGAG 700
C12C                            TACGGTAGGTGTCTTTGCGTGTGTGTCTGTAGTTATAGTATTCTAACGAG 700
Madina_2008_Ser_homozygote      TACGGTAGGTGTCTTTGCGTGTGTGTCTGTAGTTATAGTATTCTAACGAG 700
Direct_seq_Ser_homozygotes      TACGGTAGGTGTCTTTGCGTGTGTGTCTGTAGTTATAGTATTCTAACGAG 700
                                ****************** ******  **  * * ***************

C11A                            GTGCTCTTCTTCCCATCACTTCTTGGGAGTCAGCTGGGTGCGGG 744
C11B                            GTGCTCTTCTTCCCATCACTTCTTGGGAGTCAGCTGGGTGCGGG 744
C11C                            GTGCTCTTCTTCCCATCACTTCTTGGGAGTCAGCTGGGTGCGGG 744
C12A                            GTGCTCTTCTTCCCATCACTTCTCGGGAGTCAGCTGGGTGCGGG 744
C12B                            GTGCTCTTCTTCCCATCACTTCTTGGGAGTCAGCTGGGTGCGGG 744
C12C                            GTGCTCTTCTTCCCATCACTTCTTGGGAGTCAGCTGGGTGCGGG 744
Madina_2008_Ser_homozygote      GTGCTCTTCTTCCCATCACTTCTTGGGAGTCAGCTGGGTGCGGG 744
```

```
Direct_seq_Ser_homozygotes      GTGCTCTTCTTCCCATCACTTCTTGGGAGTCAGCTGGGTGCGGG 744
                                *********************** ********************
```
